# What’s in our bin? Labs kick off and demand the transition towards a circular economy for lab plastics

**DOI:** 10.1038/s44319-024-00360-x

**Published:** 2025-01-06

**Authors:** Philipp M Weber, Cleophea Michelsen, Melina Kerou

**Affiliations:** 1 Archaea Biology and Ecogenomics Unit, Department of Functional and Evolutionary Ecology, Faculty of Life Sciences, University of Vienna, Djerassiplatz 1, 1030 Vienna, Austria; 2 Merck KGaA, Frankfurter Strasse 250, 64293 Darmstadt, Germany; 3 Present Address: EMBO, Heidelberg, Germany

**Keywords:** Economics, Law & Politics, Evolution & Ecology, Science Policy & Publishing

## Abstract

An analysis by Green Labs Austria shows the amount and content of plastic waste generated by life science research. To reduce waste and help to address global plastic pollution, labs should proactively explore alternatives and recycling methods.

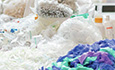

Maria is focused on transferring a pellet containing precious *Lokiarchaeota* cells—this fastidious microorganism recently revolutionized our view of early eukaryotic evolution—to a new, sterile falcon tube after centrifugation. She needs to repeat this multiple times to increase the biomass for omics experiments. But not only the cell mass increases. Looking to her right, Maria sees the bin overflowing with used plastic tubes and pipettes.

Experimental biomedical research crucially relies on single-use plastics. Easy to use and consistent, plastic products have permeated daily lab routines and have made research easier and more efficient. However, this comes at the cost of contributing to the global plastic crisis as research labs generate millions of tons of plastic waste each year (Urbina et al, 2015).

## The need to address plastic pollution

Plastic pollution has been identified as one of the top ten global environmental challenges by the UN Environment Programme (UNEP). Since the 1950s, ~8.3 billion tons of plastic have been produced, 6.3 billion tons of which ended up as waste (Geyer et al, 2017). Most of this has been incinerated, releasing harmful emissions, buried in landfills, or discarded into the environment. As most plastic materials are not biodegradable, the waste accumulates in the soils and the oceans, choking marine wildlife and poisoning the groundwater. According to UNEP, only a systemic shift from a linear to a circular economy can end the plastic crisis. But although time is scarce, we are merely at the beginning of transforming markets and behaviors towards this goal. Here, we highlight how life scientists can contribute to the solution, first by closely examining their laboratory waste and by engaging with key stakeholders. Such efforts can start discussions on circular principles relevant to academic labs and, potentially, other sectors, such as healthcare and the life-science industry.

“As most plastic materials are not biodegradable, the waste accumulates in the soils and the oceans, choking marine wildlife and poisoning the groundwater.”

What is a circular economy and how can labs become part of it? Today’s linear economy is dominated by the make-use-waste pattern. We take materials from the Earth, turn them into products, and eventually throw them away. In most cases, this means introducing non-natural substances into the environment and releasing greenhouse gas emissions all along the way. By way of example, the polypropylene (PP) falcon tube that Maria uses originates thousands of kilometers away from Vienna. Crude-oil is extracted in countries such as Norway or Saudi Arabia and transported to oil refineries that produce millions of tons of raw materials including PP and polyethylene (PE) that are the feedstocks to manufacture high-tech plastics such as the falcon tube. In Austria, the falcon tube might end up at an incineration plant with heat recovery, which generates 1.36 kg CO_2_equivalent/kg PP among other toxic combustion products. If we add the energy-intense production and transport, the carbon footprint of the tube will be multiple times this amount (Ragazzi et al, 2023).

It is therefore essential to accelerate the transition to a circular economy where no longer needed products are transformed into new ones. This huge undertaking requires a systemic change with closed-loop resource management (Kara et al, 2022) based on the so-called R-strategies: Reduce, Reuse, Recycle and Repurpose. Using fewer products longer is the single most important lever for addressing plastic pollution in a timely manner. Data-driven modeling approaches have shown that combined strategies of reduction, recycling, design for recycling and substitution with alternative materials can reduce plastic pollution by up to 50% until 2040 compared to business-as-usual scenarios, while additional upstream and downstream interventions—such as waste management improvement and scale-up—could increase this reduction to up to 80% (Lau et al, 2020).

## Reuse, recycling, and replacements

Pilot efforts in a laboratory context have illustrated that reuse and substitution, along with optimizing experimental protocols, could significantly reduce plastic waste (Alves et al, 2021; Kilcoyne et al, 2022; Estevez-Torres et al, 2024; Freese et al, 2024). Common calculations about the CO_2_ equivalent footprint of replacement and re-use strategies for laboratory single-use plastics typically revolve around water and energy usage, as well as personnel costs and time investment. However, a recent life cycle assessment—a process of evaluating the impacts that a product has on the environment over the entire period of its life—of single-use plastics versus re-use of plastic or use of glass items revealed an up to an 11-fold reduction in CO_2_ equivalent in the case of re-use scenarios, while incurring similar or even lower costs over time (Farley and Nicolet, 2023).

“Pilot efforts in a laboratory context have illustrated that reuse and substitution, along with optimizing experimental protocols, could significantly reduce plastic waste.”

Not all laboratory plastics can be substituted with alternative materials. However, emissions reduction can be achieved by eliminating carbon-heavy steps during the production and end-of-life processes or by mixing polymers with non-oil-based materials such as natural fibers to create biocomposites. For example, used cooking oil (UCO) offers an alternative to crude oil in the synthesis of high-quality polymers (PP-UCO), with the potential to reduce product emissions by up to 48% by avoiding raw material extraction (Ragazzi et al, 2023). Despite this potential, lab products made from UCO or biocomposites are still rare, with centrifuge tubes and qPCR plates among the few examples. The limited availability of biological source materials and the need for novel technical processes likely limit their commercial viability. Another barrier is the challenge of meeting purity, consistency, and stability requirements. While lower-emission biodegradable plastics can degrade under certain conditions (Chen et al, 2024), they and biocomposites do not meet the standards for many lab products. However, they could easily be used for packaging and applications with less stringent requirements.

**Figure 1:**
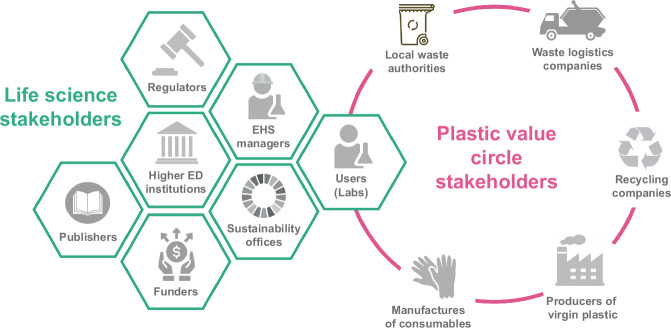
Intersecting life science and plastic stakeholders. Relevant stakeholders of the life science system are shown in the honeycombs on the left (green) and along the plastic value circle on the right (pink).

The next step after reduce and replace would be recycling. In the EU’s Waste Framework Directive, it is defined as any recovery operation by which waste materials are reprocessed into products, materials or substances whether for the original or other purposes. Closed-loop recycling would avoid most emissions associated with the current production, use, and disposal of plastic in laboratories. In theory, all plastic types are recyclable (see infobox); however, different properties, such as density and softness, require different methods. For example, only 10–15% of high-density PE is recycled in Europe because it needs to be separated in a laborious manner before treatment. This highlights the need for a systemic recycling approach, whereby all actors along the value chain of a material are contributing. In the case of single-use plastics for labs, stakeholders in the life sciences and industry overlap: the producers of plastic raw material (typically petrochemical companies), the manufacturers of consumables, the users (laboratory personnel), local waste authorities, waste logistics companies and recycling companies. Within the life sciences, we have institutional waste managers or Environmental Health and Safety (EHS) managers, sustainability offices, institutions, funders, publishers, and regulators (Fig. 1).

Although life scientists are increasingly concerned about the environmental impact of their work (Farley, 2022; Winter et al, 2023), few attempts have been made to revise laboratory waste management concepts towards recycling. The current fate of lab plastics depends on the type of biological or chemical substances they have been in contact with and the biosafety level of the laboratory; they are usually either disposed of in a landfill or incinerated, often with an energy-intensive autoclaving step beforehand. Items that were in contact with toxic or biologically sensitive material from biosafety level 2 or higher are collected separately and handled by specialized waste management companies, which transport them in safety containers to appropriate incineration facilities. The environmental impact of incineration, however, with or without energy recovery, is significant and has been shown to contribute 28–54% to the total emissions of selected laboratory items made from the most common plastic polymers: up to 2.57 kg CO_2_ eq/kg waste or 1.06-1.8 in the case of incineration with energy recovery (Ragazzi et al, 2023).

Nevertheless, there is a growing appreciation that a large proportion of laboratory plastic waste, especially from biosafety level 1 laboratories, fulfills the criteria for recycling. A small number of attempts at developing recycling processes have been reported, in all cases a result of initiatives from laboratory scientists themselves (Freese et al, 2024; Kilcoyne et al, 2022; Kuntin, 2018; Green Labs Austria recycling pipeline, 2021). Upscaling of these pilot projects, however, is virtually impossible at the moment, owing to the vast differences in waste management and recycling regulations across countries and even municipalities and the diverse requirements of private recycling plants. In addition, such initiatives require institutional support and alignment of a number of actors, such as EHS managers and infrastructure administrators, which is not always present.

The recycling potential is underlined by the emergence of take-back schemes for selected items introduced by manufacturers. Several startup companies are trying to develop low-emission methods for decontaminating and recycling laboratory plastics. For example, LabCycle, RecycleLab, and Polycarbin claim to increase the recycling of non-hazardous PS, PP, and Polyethylene terephthalate (PET). Polybcarbin offers lab products with 20-92% of recycled plastics. Moreover, established consumable producers such as Merck KGaA have started to engage with their customers to develop a plastic-waste assessment.

**Figure 2:**
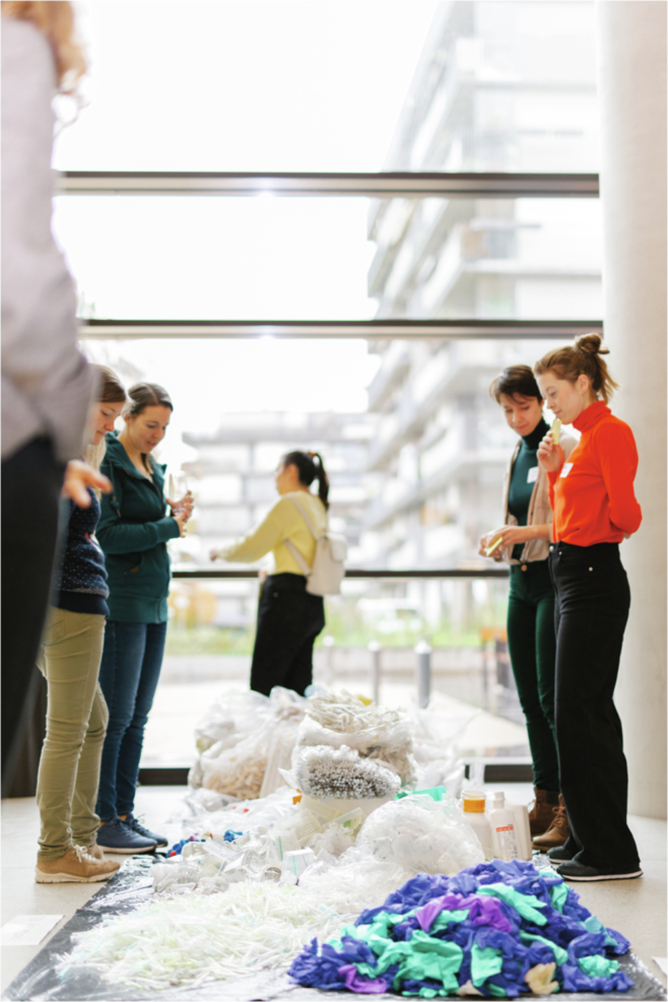
A pile of plastic waste. Participants of the workshop “The sustainable lab of the future: rethinking single-use plastics” that was organized by Green Labs Austria, Merck KGaA, and the University of Vienna in November 2022 look devoutly at the plastic waste produced by their labs.

As the main users of these products, researchers have a central and powerful role, which should be harnessed in demanding the necessary transition towards a circular economy of laboratory plastics. To this end, Green Labs Austria and Merck KGaA organized a workshop at the University of Vienna in November 2022 with representatives from across the single-use plastics value chain, and with stakeholders from the University of Vienna (Fig. 2). The starting point for this workshop were two major questions: What is in the bins, and what role can labs, universities or other institutions play in creating a circular economy for lab plastics?

**Infobox: most common plastic types used in labs**

**Table:** 

Plastic type	Name	Recyclability
PP	Polypropylene	Highly recyclable but requires pure-type collection
PS	Polystyrene	Generally recyclable but limited due to low density
PE	Polyethylene	Highly recyclable but needs to be sorted from harder plastics
PET	Polyethylene terephthalate	Highly recyclable but requires pure-type collection

Source: EuRIC - Plastic Recycling Factsheet.

## What is in our lab bin?

There is currently no coherent and pragmatic concept of what a waste-free lab could look like. The first step therefore is to understand the components, volumes and contaminants of laboratory waste. Only a few such studies are available (Urbina et al, 2015; Freese et al, 2024; Alves et al, 2021; Kilcoyne et al, 2022), of which the only quantitative data widely propagated in the literature comes from a correspondence by Urbina et al from the University of Exeter in 2015, stating that 280 bench scientists generated roughly 267 tons of plastic during a year (Urbina et al, 2015). While this article started the much-needed discussion within the scientific community regarding the use of single-use plastic, there are limitations in the assumptions and generalizations that can be drawn from it, as it was not meant to provide analytical data on the quality, quantity and types of laboratory plastic waste. Closing data gaps by investigating plastic waste production in different laboratories and providing quantitative data in an adequate resolution is critical to further collaborate along the value chain with raw material producers, manufacturers, users and recyclers for a waste-free circular lab.

**Figure 3:**
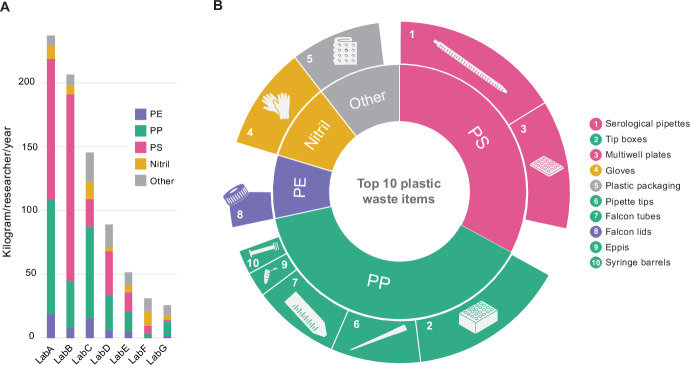
The quantity and quality of lab plastic waste produced by different science labs. (**A**) Amounts of plastic waste and plastic types produced by seven labs (Lab A–G). For comparability the amount was normalized to the annual production in kilogram per researcher. The total waste of one week was divided by the people that worked in lab and their working hours. We assumed a 30-hour work week and 50 weeks per year. The plastic types PP, PS, PE, Nitrile and other (the latter referring to mixed plastic fractions such as packaging material) are indicated. (**B**) Stacked donut plot shows in the inner circle the fraction of the different plastic types (PP 39%, PS 33%, PE 8%, Nitril 10% and Other 10%) of five labs (in two of the seven laboratories, the waste was melted together during autoclaving and we were unable to determine the weight of the individual items) (Lab A, C, E, F, and G). The outer circle shows the contribution of the ten items that contribute to most weight: (1) Serological pipettes (16.1%), (2) Tip Boxes (15.0%), (3) Multiwell plates (12.2%), (4) Gloves (9.8%), (5) Plastic packaging (8.6%), (6) Pipette tips (8.5%), (7) Falcon tubes (8.2%), (8) Falcon lids (3.4%), (9) Eppendorf type tubes (2.3%), (10) Syringe barrels (2.3%). All data can be shared upon request.

To initiate this process, Green Labs Austria analyzed the plastic waste produced by seven labs in different research fields during a working week, excluding plastic from biohazardous or toxic waste (Fig. 1). Most laboratory items were made from three different types of rigid plastics: polystyrene (PS), PP, and PE, and elastic nitrile, which is used to make gloves. In all, 74% of the waste was composed of PS and PP (42% PS and 32% PP, respectively), while 13% of the weight was composed of PE and nitrile (both between 6-7%), with the remaining 13% being a mixed plastic fraction composed of e.g., packaging. By extrapolating the annual waste production in kilograms per researcher, assuming 30 h at the bench during a normal work week, we calculated that a scientist produces on average 116 kg plastics per year (Fig. 3A). This figure is about nine times lower than the amount reported by Urbina et al (2015). Given the unclear source of the estimated figure in the latter it is not possible to draw further conclusions comparing these numbers. However, we maintain that our result is representative of the mean plastic waste production of a variety of life-science labs in Western countries.

“…a scientist produces on average 116 kg plastics per year.”

In addition, we found that the type and research methods of the laboratory largely determine the main methods and protocols used, and as such, can substantially affect the amount of plastic waste produced. The laboratories that participated in our study have the following backgrounds: medical research on disease models (cancer, allergy), pharmaceutical research (molecular drug targeting, drug delivery, biophysics), biotechnology and bioprocess engineering. The waste production ranged from 32.3 kg to 236.9 kg/researcher/year. The lowest amounts were produced by laboratories mostly focusing on standard molecular techniques, such as nucleic acid purification and amplification, cultivation of microorganisms, and protein biochemistry. In contrast, the highest amount was generated in laboratories that employ tissue and cell culture-based methods (Fig. 3A, Lab C-G and A-B, respectively). Surprisingly, ten standard laboratory items commonly used in all laboratories are responsible for 86.6% (w/w) of the waste; serological pipettes, pipette tips and boxes, multi-well plates, centrifuge tubes and gloves (Fig. 3B). These products would have the biggest impact on waste reduction if they were produced from alternative materials or non-fossil fuel-based monomers and designed for recycling into high-quality laboratory items of equal value. Tip boxes, ranked as the second largest contributor, present an immediate opportunity for reduction through reuse or recycling programs.

“…the highest amount (of plastic waste) was generated in laboratories that employ tissue and cell culture-based methods.”

It should be noted that these waste fractions included autoclaved waste that had been in contact with non-pathogenic microbial cultures and nucleic acids (Biosafety level 1). While it is clear that any waste management plan needs to comply with EHS protocols, our results and interactions with recycling partners showed that, when proper sorting and decontamination processes are in place, the majority of laboratory plastic from Biosafety level 1 laboratories can be safely channeled into recycling processes.

“…the majority of laboratory plastic from Biosafety level 1 laboratories can be safely channeled into recycling processes.”

## Filling data gaps as a starting point towards a circular economy

Collecting data on single-use plastic waste from labs in different countries and on current disposal routes and regulations is a necessary starting point for developing a general concept for a circular economy for plastics. This information is essential for formulating clear demands towards other stakeholders, including the demand to prioritize sustainability, to re-think the design, production and use of laboratory items, to conduct and share appropriate life-cycle assessment studies on products, and assume a more active role in working towards a circular economy.

Although we are only at the beginning of data collection, three conclusions can already be drawn: First, filling data gaps on plastic waste should be the starting point for working on circular solutions and understanding the role of the laboratory in circular transformation. Second, significant waste reduction could be achieved by using alternative materials not based on fossil fuels for the main plastic items identified in this study and by developing recycling streams for them. Lastly, we need an updated framework for the classification of laboratory waste and a review of the relevant legal framework.

## What role can labs and institutions play in creating the circular lab?

Systemic change in laboratory waste management requires collaboration and alignment of all actors in the plastics value chain. To ensure the representation of all sectors involved, the workshop participants included laboratory researchers in universities and research institutes, professors, institute directors and senior management, manufacturers of research consumables, waste managers, and sustainability professionals. Their inputs and intensive discussions inspired the following concrete suggestions for labs and research institutes/universities.

Laboratories are spaces for discussion and experimentation, including questions related to the environmental impact of research. They also serve as hubs for innovation, where novel approaches to reducing environmental impacts can be tested, refined, and integrated into standard practices. Laboratories provide unique opportunities to use the campus as a testbed for addressing real-world challenges, embodying the concept of a ‘living lab’ as pioneered by the Massachusetts Institute of Technology (MIT Living Labs). They also have the scientific background to conduct this research and the means to efficiently disseminate their findings to the wider community. Importantly, rethinking protocols—such as optimizing a sequencing procedure—can not only reduce waste, time and costs but also lead to higher reproducibility. Lastly, scientists’ findings carry credibility within academia and beyond.

The role of research institutes and universities is to provide a clear sustainability strategy and goals along with the necessary resources, infrastructure, and implementation processes, especially for waste managers, EHS managers, and so on. Regarding the single-use plastic challenge, universities can be used to develop circular recycling solutions on a small scale, demonstrating, for example, the feasibility of recycling plastics from labs. Characterizing the different waste streams and collaborating with other institutions and companies along the value chain can inspire other sectors, like the carbon-heavy health sector. Furthermore, universities should introduce measures transparently, discuss best practices, and revise projects when new knowledge is gained. For example, when recycling quotas are introduced, this should always be accompanied by a critical assessment of what recycling means and how transparent the process is, and any implementation must be regularly evaluated. Finally, universities should create a research culture that rewards sustainability efforts, such as reducing the amount of single-use plastics.

In fact, life-science research has already made tangible contributions to addressing the plastic crisis, notably the recycling of petroleum-derived polymers that are recalcitrant to natural biodegradation. One outstanding example is the search for and optimization of plastic-degrading enzymes. This led to the discovery of *Ideonella sakaiensis*, a bacterium capable of utilizing PET as its primary carbon and energy source (Yoshida et al, 2016). Crucially, the PETase involved breaks down PET into its monomers, which can be recycled to create new plastics. This approach is now being scaled to industrial levels: in May 2024, construction began on a PET biorecycling plant in France with a target capacity of 50 kilotons per year (Carbios).

## Global incentives and conclusions

The rising global awareness of the detrimental effects of climate change and environmental pollution is slowly but surely translated into a variety of legislative and regulatory tools to facilitate and accelerate transformation to a sustainable economy. Among these, the resolution adopted by the UN Environmental Assembly to work towards an international legally binding agreement to End Plastic Pollution is expected to mobilize all stakeholders and address the full lifecycle of plastics. Additionally, the adoption of the Corporate Sustainability Reporting Directive by the EU in January 2023, under which a number of research institutions will also be required to submit regular reports on the societal and environmental impact of their operations, provides a strong incentive for developing sustainability strategies. International funders and other stakeholders recently addressed the lack of sustainability commitments in funding activities and published the Heidelberg Agreement on Environmental Sustainability in Research Funding (Weber et al, 2024). Some funding bodies have already started implementing sustainability considerations into their schemes, for example, by requesting statements for the anticipated environmental impact of research project proposals and respective reduction and resource-conserving measures (e.g., DFG) or a minimum level of lab certification programs, like LEAF or My Green Lab (see policies by Wellcome and CRUK). Institutional pledges and campaigns to achieve net-zero status and/or eliminate single-use plastic on campus are also gaining momentum (e.g., Race to Net Zero, Uni Leeds, ETH, University of Vienna). As a significant proportion of Scope 3 CO_2_ equivalent emissions of research institutes are connected to laboratory operations (e.g., ETH whitepaper and Allea report), it is anticipated that the need to develop circular solutions will involve the reduction of the use of single-use plastics.

To conclude, research can not only contribute to sustainability goals by addressing relevant scientific questions, but also by challenging the way science is done. Labs should initiate and drive the reduction of single-use plastics made from fossil fuels at the bench level by demanding new products and developing and publishing new protocols. But to fulfill this role they need resources, guidance and a clear strategy from universities and institutions.

“Labs should initiate and drive the reduction of single-use plastics made from fossil fuels at the bench level by demanding new products and developing and publishing new protocols.”

The formation of cross-stakeholder networks, such as the collaboration between Green Labs Austria and Merck, is an essential approach to accelerate the necessary systemic changes to achieve a circular economy. Similar initiatives have emerged in other fields such as Chemistry, where networks dedicated to disseminating and applying the principles of Green Chemistry are fostering cross-stakeholder collaboration to instigate systemic change (e.g., Beyond Benign). It is imperative for the research community to acknowledge and address the environmental impact of their activities. Moreover, researchers need to find ways to appropriately employ their collective knowledge to address the pressing challenges of our time, such as using molecular biology for plastic-degradation technologies. Only then can we contribute adequately to the necessary transformations towards sustainability.

## Supplementary information

Peer Review File
