# Supplementary material for: What’s in our bin? Labs kick off and demand the transition towards a circular economy for lab plastics
Source: EMBO Rep. 2025 Jan 6;26(2):297–302. doi: 10.1038/s44319-024-00360-x (PMC11772835; doi:10.1038/s44319-024-00360-x)
Supplement: Supplementary file 1 — Peer Review File [file 44319_2024_360_MOESM1_ESM.pdf]

EMBO reports

What's in our bin? Labs kick off and demand the transition towards a circular economy for lab plastics

Philipp Weber, Cleophea Michelsen, Melina Kerou, and Philipp Weber

Corresponding authors: Philipp Weber (philipp.weber@univie.ac.at) , Melina Kerou (melina.kerou@univie.ac.at)

Review Timeline:

Submission Date:	8th Dec 24
Accepted:	18th Dec 24

Editor: Holger Breithaupt

Transaction Report:

This article was editorially reviewed.
